# Pragmatic Profiles of Adults with Fragile X Syndrome and Williams Syndrome

**DOI:** 10.3390/brainsci12030385

**Published:** 2022-03-13

**Authors:** Eliseo Diez-Itza, Aitana Viejo, Maite Fernández-Urquiza

**Affiliations:** LOGIN Research Group, University of Oviedo, 33003 Oviedo, Spain; viejoaitana@uniovi.es (A.V.); fernandezmaite@uniovi.es (M.F.-U.)

**Keywords:** fragile X syndrome, Williams syndrome, pragmatic impairment, pragmatic assessment, Gricean maxims of conversation, social (pragmatic) communication disorder

## Abstract

Linguistic phenotypes of individuals with Fragile X (FXS) and Williams (WS) syndromes exhibit various degrees of pragmatic impairment, involving difficulties in social communication and in adapting to conversational principles. The goal of the present study was to explore syndrome-specific pragmatic profiles of adults with FXS and WS based on the assessment of the observance of Gricean maxims of conversation. The participants were 12 Spanish-speaking adults (6 FXS/6 WS), without a diagnosis of ASD, whose extensive naturalistic conversations (71,859 words) were transcribed and coded with the CHILDES/TALKBANK tools and the PREP-CORP pragmatic protocol. Violations of the maxims of conversation were analyzed, and indexes of cooperation and conversational response were obtained. Both groups showed reduced verbal production and repetitive dysfluencies; prominent features in the FXS profile were higher proportion of non-contingent language, perseverations of topic and form, and impulsive conversational responses; in the WS profile, salient characteristics were higher proportion of tangential utterances, reformulations, and conversational responses reflecting overly literal interpretation. Pragmatic profiles of violation of conversational maxims reflect specific communication skills impaired in adults with FXS and WS and raise the need for assessment and intervention methods that specifically address their social communication abilities.

## 1. Introduction

Fragile X syndrome (FXS) and Williams syndrome (WS) are neurodevelopmental genetic disorders characterized by specific phenotypical profiles of relative strengths and weaknesses in the neurocognitive domain, including intellectual disability and a range of behavioral problems [1,2,3,4,5].

FXS is the most common identified cause of inherited intellectual disability. It is caused by the mutation of the Fragile X Mental Retardation gene (FMR1) at the chromosome Xq27.3 and the consequent lack of the Fragile X Mental Retardation Protein (FMRP), which is predominantly expressed in neurons [2].

WS is a rare neurodevelopmental disorder caused by the heterozygous deletion of 25–27 genes at chromosome 7q11.23, which has a complex genomic architecture, and its multisystemic phenotype is characterized by mild to moderate intellectual disability, cardiovascular disease, and distinctive facies [6].

As expected for an X-linked disorder, males with FXS tend to exhibit more cognitive and behavioral problems relative to females with FXS, and most of the research on FXS has targeted males [7]. Conversely, in the case of WS, there is no evidence of gender differences in the cross-cultural behavioral profiles [8].

The phenotypical outcomes have implications for language profiles in both syndromes, which present within-syndrome and cross-syndrome differences. Within-syndrome analyses typically display uneven profiles of relative strengths and weaknesses. In parallel, cross-syndrome analyses reveal profiles with shared and syndrome specific features [1,9,10,11]. Language profiles of males and females with FXS present similar types of language learning problems, but with a difference in degree, as has been observed in repetitive language [11,12]. Vocabulary is described as a relative strength, including lexical diversity of language production. In turn, morphosyntactic production shows relative weakness in complexity and delays in narration and conversation that exceed non-verbal mental age (NVMA) expectations in males. Receptive vocabulary and grammar tend to be commensurate in FXS with NVMA during adolescence and young adulthood [9]. Within the neurocognitive profile of older children and adolescents with WS, language was initially considered a strength and even relatively preserved and dissociated from cognition [1]. However, only receptive vocabulary has been reported better than predicted by NVMA, while phonology, grammar, and vocabulary remain as syndrome specific areas of relative strength [13].

Despite marked differences in social orientation, FXS and WS share asynchronous profiles of pragmatic weakness that arise in social communication contexts [9,10,11]. Hyperarousal and social anxiety have been pointed to in explanations of pragmatic impairments in FXS, such as perseverative and tangential language or reduced and dysfluent speech that cannot be accounted for by level of intellectual functioning or autism [14]. Individuals with WS also exhibit a personality with an undercurrent of anxiety related to social situations, despite high sociability, overfriendliness, and empathy [3]. Links between anxiety and social behavior have been related to impairments in executive functioning [15].

Pragmatic skills have been investigated independently in both syndromes or in contrast with other neurodevelopmental disorders, such as Down syndrome, in search for specific profiles [16], but to our knowledge there are no previous comparisons of the pragmatic profiles of FXS and WS; therefore, the present study was designed to explore common and syndrome-specific characteristics in the pragmatic profile of these two syndromes.

### 1.1. Pragmatic Impairment across Neurodevelopmental Disorders

Pragmatic impairment was primarily considered a symptom of autism in early studies, although it was soon differentiated from autism and included, within the Language Development Disorders, as a Semantic-Pragmatic Disorder (SPD) when it occurred “without autism” [17]. SPD was characterized by features such as excessive verbal production, incoherent discourse, atypical vocabulary, or inadequate conversational skills. It was later redefined as Pragmatic Language Impairment and was explored in the borderlands of ASD and Specific Language Impairment (SLI), as well as in its comorbid occurrence in genetic neurodevelopmental disorders entailing intellectual disability [18,19,20].

Finally, it was included in the latest version of DSM-5 as the diagnostic category of “Social (Pragmatic) Communication Disorder (SPCD)” [21]. SPCD is characterized by socially inappropriate use of communication, language not adapted to the context and the needs of the interlocutor, difficulties in following the rules of conversation and narration, and difficulties in understanding non-literal language [22]. However, in the DSM-5, SPCD is presented as an independent diagnostic category, which has been considered problematic, especially with regard to its relationship with ASD, raising controversy and posing dilemmas to diagnosis, treatment, and research [23]. For these reasons, it is suggested that SPCD would better describe a cross-sectional profile of different neurodevelopmental disorders [24,25].

### 1.2. Pragmatic Skills in FXS and in WS: Narratives and Conversation

Individuals with FXS and WS exhibit pragmatic difficulties in social interaction and communication, including problems in maintaining a conversation topic, perseveration, repetitive language, impulsivity, and inappropriate responses [26]. Research on pragmatic skills is scarce and has relied on indirect reports [20] and on the assessment of narratives and conversations [27], which point to syndrome specificities.

Narrative skills are a crucial aspect of social communication and provide a means for investigating aspects of the microstructure (i.e., average utterance length, narrative length, syntactic complexity) and macrostructure (i.e., evaluation, story structure, thematic maintenance) of discourse as it is produced in natural contexts. Those aspects of narratives have been addressed in several studies of individuals with WS or FXS alone or compared with other populations with neurodevelopmental disorders, such as Down syndrome, revealing specific weaknesses in macrostructure aspects (i.e., thematic structure) [28,29,30,31,32,33,34,35,36].

Even more scarce are the studies on conversational discourse both in FXS and in WS. In one of the first conversational analyses of males with FXS, Sudhalter et al. [37] reported deviant repetitive language that was interpreted as etiologically specific, as it was distinct from that of males with Down syndrome or autism. Repetitive language included: perseveration (phrasal, sentential, and topical), jargoning, echolalia, and affirming by repetition. Deviant repetitive language was distinctly different in FXS, where perseveration was pervasive (86%), compared with autism, where echolalia predominated (79%). In an additional study, Sudhalter et al. [38] confirmed that perseverative language in FXS was not a correlate of a syntactic deficit but that it was a syndrome-specific feature revealing an asynchronous development of language components. Ferrier et al. [39] observed differences in the patterns of conversation of individuals with FXS, autism, and Down syndrome based on use of self-repetitions and inappropriate responses violating multiple conversational rules.

In addition to perseverative speech, individuals with FXS exhibit tangential language, defined as personal and highly idiosyncratic associations of words, phrases, or topics that are not pertinent to the thread of conversation [40]. Dysfluent speech has also been described as characteristic of individuals with FXS—in particular, stuttered dysfluencies (i.e., repetitions of sounds, words or sentences), as opposed to nonstuttered dysfluencies (i.e., self-corrections, reformulations, or false-starts) [14]. Noncontingent discourse (i.e., inappropriate in quality topic maintenance and topic change) was found more frequently in children and adolescents with both FXS and autism, whereas perseveration appeared to be a defining feature of FXS regardless of autism status [41]. Furthermore, there have been reported diverse effects of the sampling context (conversational vs. narrative), together with autism symptom severity, on topic perseveration, talkativeness, length of communicative units, lexical diversity, fluency, and intelligibility [42,43].

In contrast with FXS, social cognition of adolescents with WS was described initially as a strength, as they showed awareness of listener needs, engagement to the listener, and affective expression in narratives [44]. Their conversational style was considered hyperverbal, showing what was referred to as “cocktail party speech”: superficial, stereotyped and “odd” language, previously observed in hydrocephalic children. However, the first analyses of quantity and quality of conversations of children and adolescents with WS showed that only a substantial minority of the participants with WS presented such profile, including inappropriate clichés, idioms, social phrases, fillers, irrelevant personal experiences, false starts, and stallers [45].

Subsequent analyses of conversations of individuals with WS indicated that more than 25% of the utterances were inappropriate, which in half of the cases were due to problems of expressive syntax and semantics and insufficient quantity of information provided [46]. Social communication deficits, in the form of atypical social interaction (conversation exchange structure, turn taking, and information transfer) and conversational inadequacy, were also reported and linked to low cognitive abilities [47]. In a scale of social responsiveness, parents of adults with WS reported strengths in social motivation, contrasting with moderate to severe impairments in social cognition; parents emphasized that individuals with WS needed support in initiating and maintaining conversations [48].

### 1.3. Pragmatic Skills and Autistic Symptoms in FXS and WS

Pragmatic difficulties in social interaction and communication are often described as autistic symptoms in individuals with intellectual disabilities of genetic origin [49,50]. Autism spectrum disorder phenomenology, including pragmatic impairments, is a major component of the FXS phenotype, present in more than 90% of cases in males [51]. Overlapping phenotypes between FXS and ASD entail comorbid diagnosis in a widely variable proportion of cases (5–75%), depending on the breadth of the diagnostic criteria [52,53]. Although symptoms related to social-communicative deficits have been reported as the main predictors of a subsequent diagnosis of ASD in infants with FXS [54], restricted and repetitive behaviors (RRB) may play a more prominent role in distinguishing males with FXS and comorbid autism [55]. Impairments in pragmatic language and theory of mind have also been pointed out as overlapping symptoms of children with idiopathic autism and children with FXS who meet criteria for autism [56]. The trajectories of development suggest that social communication impairment is a core shared characteristic of FXS and ASD, while RRB would emerge as a more specific feature of idiopathic ASD [57].

Autistic symptoms in FXS might then differ from those of non-syndromic ASD [58]. Social communication difficulties in individuals with FXS and comorbid autism have been reported as less severe than those found in cases of idiopathic autism [59]. Severity of autism symptoms has been associated with a reduced production of utterances in conversation and a higher proportion of non-contingent speech [41,42], while a standardized pragmatic judgment subtest failed to reveal differences between boys with FXS with and without ASD [60]. Furthermore, ASD diagnosis can mask important differences among individuals with FXS and between FXS and non-syndromic ASD [61]. Given those differences, especially in social communication, categorical diagnosis of ASD may be misleading for intervention in FXS [62].

Individuals with WS were initially characterized by a profile of hypersociability and were thus considered to represent the polar opposite of autism in the social and communicative domain [63]. Only in more recent times, studies comparing pragmatic language ability indicate a number of shared characteristics between WS and ASD in social interaction and communication skills [64,65,66,67]. Despite the opposite trajectories of social development in the two disorders, they share analogous disruptions in social cognition, while differences arise in social motivation, which is reduced in ASD and enhanced in WS [68]. In the same vein, adaptive functioning profiles of young children with WS compared with their peers with ASD are at a similar level, although those with WS show relative strengths in socialization [69]. Social cognition has also been related to the perception of emotions, where WS children experience greater difficulties identifying emotions than children with ASD [65,70]. However, young children with WS and limited language presented fewer socio-communicative abnormalities than children with ASD [71].

ASD comorbid diagnosis in neurodevelopmental disorders may have clinical utility to characterize phenotypic variability, although it is partially based on pragmatic impairments that are not clearly differentiated from those also observed in FXS without autism. If RRB-like features are not present, a more accurate characterization of pragmatic impairment in many individuals with intellectual disability of genetic origin might be provided by DSM-5 diagnosis of Social (Pragmatic) Communication Disorder (SPCD) [72].

### 1.4. Assessment of Pragmatic Impairment

The assessment of the pragmatic profile in individuals with intellectual disability is frequently oriented to comorbid diagnosis of ASD, especially in the case of FXS. Therefore, it is based in many cases on instruments designed for autism screening, such as the ADOS-2, ADI-R, Autism Screening Instrument for Education Planning (ASIEP-3), Social Communication Questionnaire (SCQ), and the Social Responsiveness Scale (SRS-2) [51,52,73,74,75]. Instruments for the assessment of pragmatic impairment, such as the Children Communication Checklist (CCC), have also been used in children and young adults with FXS and WS [20,56,66]. Formal tests of pragmatics are rare, as the use of language in natural situations of social interaction is not directly captured in such controlled contexts of assessment [76].

Oral language sample analysis is considered the most valid procedure for pragmatic evaluation [77], such as expressive language sampling [78], specifically oriented to the study of FXS, or clinical discourse analysis (CDA) [79]. CDA is based on Grice’s [80] conversational cooperation maxims: message imprecision (maxim of quality), thematic relevance (maxim of relation), redundant or informatively poor utterances (maxim of quantity), and problems with the form of conversational turns (maxim of manner). CDA has also been used with brain-damaged adults, leading to procedures such as La Trobe communication questionnaire [81]. Different versions of a Conversational Violation Test based on Gricean maxims have been used to assess pragmatic impairment in children with ASD and with SLI, and in TD children and adults [82,83]. Additionally based on Grice’s maxims is the Pragmatic Evaluation Protocol for Corpora (PREP-CORP) [84] that has been used for the study of neurodevelopmental genetic syndromes [34,85]. Through a time-intensive approach of hand coding, this instrument provides a highly detailed analysis that is considered more accurate than standard transcription procedures for the coding of natural conversation in FXS [86].

### 1.5. Objectives

Taking into account the scarcity of studies on pragmatic impairment and of instruments for its assessment in neurodevelopmental genetic syndromes, the present study had two main objectives: (1) to explore the pragmatic profiles of adults with FXS and WS based on analyses of conversations; (2) to determine the feasibility of the PREP-CORP protocol for assessing pragmatic features in speech corpora.

The PREP-CORP protocol includes a section that allows the coding and quantification of the observance of Grice’s cooperation maxims in conversational utterances. Consequently, the specific objectives of the study were focused on calculating aggregate violation indexes (AVIs) and conducting between-group and within-group comparisons of the following maxims and types of violations:

- Maxim of quality (MQL): truthfulness and adjustment to world reality.
- Maxim of relation (MRL): non-related, tangential, and perseverations of topic.
- Maxim of quantity (MQT): redundant, vague, excessive, and reduced utterances.
- Maxim of manner (MMN): repetitions, reformulations, perseverations of form, and alterations of the syntactic order.
- AVI of cooperation: aggregate index of violations of all the maxims.
- AVI of conversational response: incomprehension, literal interpretation, impulsivity, and echolalia.

Analyses were expected to yield within-syndrome differences (i.e., uneven profiles of strengths and weaknesses) in the types of violations of conversational maxims; and also, cross-syndrome comparisons were expected to show profiles with overlapping and syndrome-specific conversational features.

## 2. Materials and Methods

### 2.1. Participants

The conversational speech samples were elicited from two groups of Spanish-speaking participants: 6 adult males with FXS (mean age = 38;6; range = 31;8–56;6) and 6 adults (5 females) with WS (mean age = 23;11; range = 18;7–35;5). The total size of the speech samples collected was 71,859 words (FXS = 38,555; WS = 33,304). The participants belong to a larger sample from the SYNDROLING Project [87]. Inclusion criteria for the present study were a genetic diagnosis of FXS or WS and a diagnosis of mild-to-moderate intellectual disability (ID) following DSM-5 criteria on the basis of daily skills, including the ability to participate in extended conversation; the exclusion criterion was a clinical diagnosis of comorbid ASD, including the assessment of RRBs. Individuals in the FXS group worked in a job-placement center for people with intellectual disability, while the participants in the WS group attended different educational and occupational centers. Informed consent was obtained both from the job-placement center managers and from the legal guardians of the participants.

### 2.2. Instrument

The transcripts were coded using the PREP-CORP Pragmatic Evaluation Protocol [84], adapted from the Quick Protocol for Pragmatic Assessment (PREP) [88]. PREP protocols include three sections: enunciative, textual, and interactive pragmatics. PREP was designed to be annotated by clinicians during the sessions. The PREP-CORP adaptation was devised to perform pragmatic analyses in speech corpora based on a system of labels. The items in the section of enunciative pragmatics of the PREP-CORP were applied (Appendix A). These items refer to Grice’s principle of cooperation and include as subitems or main labels the maxims of quality (MQL), relation (MRL), quantity (MQT), and manner (MMN). The PREP-CORP also allows coding for the different types of violations of each maxim in every conversational utterance using secondary labels: MQL (WOR: world reality); MRL (NRL: non-related; TNG: tangential; PER_T: perseveration of topic); MQT (RUT: redundant; VUT: vague, imprecise or indeterminate; EVP: excessive verbal production; RVP: reduced verbal production) and MMN (REP: repetition of words; REF: syntactic reformulation; PER_F: perseveration of form; ORD: syntactic order). In parallel, it allows the analysis of specific characteristics of the conversational responses using optional labels common to all the maxims (ICOM: incomprehension; LIT: literality; IMP: impulsivity; ECH: echolalia).

The following are examples of PREP-CORP coding for each of the maxims:

(a) Maxim of Quality (MQL)
  *INV: and what are you working on here nowadays?
  *PAR: on nothing.
  %xepr:$i5:MQL:IMP
(b) Maxim of Relation (MRL)
  *INV: (that cap) do you know which team it belongs to?
  *PAR: of basketball.
  %xepr:$i5:MRL:TNG:ICOM:LIT
(c) Maxim of Quantity (MQT)
  *INV: why she was called so?
  *PAR: she was called so.
  %xepr:$i5:MQT:RUT:ECH
(d) Maxim of Manner (MMN)
  *INV: what happened to the wolf and the stones?
  *PAR: and then what else? (.) and then (.) and then something else (.) and then something else (.) and then something else yet (.) and then something else yet about stones.
  %xepr:$i5:MMN:REP:PER_F

### 2.3. Procedure

Speech samples were collected in spontaneous dyadic conversations with an experienced researcher using audiovisual recordings (average duration: 45 min; range: 37–55).

A certain degree of standardization was introduced by the researcher prompting common topics to all participants, in line with the procedures developed by Abbeduto et al. [89]. The topics included school/work, favorite educators, friends and family, weekend activities, hobbies, a trip, and a visit to the doctor. The development of the topics varied across participants, following the spontaneous flow of conversation. Adults with FXS were interviewed at the job placement center and adults with WS at their home. The conversations were transcribed using the CHAT format of the CHILDES project [90]. Coding of maxim violations using PREP-CORP was conducted by two independent researchers, and the disagreements were resolved by a third researcher.

### 2.4. Data Analysis

In order to control for the differences in grammatical complexity and lexical diversity, mean length of utterances in words (MLUw) and number of different words (NDW) were calculated for both groups. The frequency of the different types of violation of the maxims was also calculated using the CLAN software from the CHILDES project. In order to control for the differences in the size of the transcripts, violation indexes (VI) were calculated: frequency of each type of violation per thousand words. The sum of the VIs provided aggregate violation indexes (AVIs) of each maxim. In turn, the four AVIs were added to obtain an AVI of cooperation (AVIofCOOP), as a global pragmatic measure. An AVI of conversational response (AVIofCONRES) was also calculated from the optional tags. The between-group and within-group differences in the VIs and AVIs were analyzed using the Student’s *t*-test for independent and related samples. In addition to significance tests, estimates of the magnitude of the observed effects were calculated according to criteria and procedures provided by Ellis [91]. There are different definitions of a standardized effect size, and the statistics can be grouped into two families: *r* family (based on correlations), and *d* family (based on mean differences). Although *d* is recommended to generalize the impact, *r* is considered a more flexible and ecologically valid statistic when the sample is small. Therefore, a multiple perspective using both *r* and *d* was adopted [92].

## 3. Results

Differences between the two groups on the previous control variables of grammatical complexity (MLUw) and lexical diversity (NDW), assessed with *t*-tests, were not statistically significant: MLUw (*t* = 1.502; *p* = 0.164); NDW (*t* = 1.544; *p* = 0.154).

Table 1 shows the indexes for the different types of violation of the maxims (VIs) and the aggregate violation indexes (AVI) for each maxim. It also includes a global measure of violation of the cooperation (AVIofCOOP) and the results of between-group *t*-test comparisons. The FXS group presented a significantly higher proportion of violations than the WS group in the maxim of relation (MRL) and, specifically, in the topic perseverative utterances (PER_T). Concerning the types of violation of the maxim of manner (MMN), the FXS group presented a significantly higher proportion of violations in perseverations of form (PER_F), while the WS group presented a significantly higher proportion of violations in reformulations (REF). No significant between-group differences were observed in the aggregate measure of violation of the cooperation (AVIofCOOP).

In the types of violations of the MRL (NRL: non-related utterances, TNG: tangential utterances, PER_T: perseveration of topic), within-group comparisons yielded the following statistically significant differences: in the FXS group, NRL < TNG (*t* = −3.229; *p* = 0.023; *d* = −1.986; *r* = −0.704) and NRL < PER_T (*t* = −4.963; *p* = 0.004; *d* = −2.356; *r* = −0.762); in the WS group, NRL < TNG (*t* = −6.140; *p* = 0.002; *d* = −4.242; *r* = −0.904) and NRL < PER_T (*t* = −3.348; *p* = 0.020; *d* = −2.474; *r* = −0.777).

In the types of violations of the MQT (RUT: redundant; VUT: vague, imprecise, or indeterminate; EVP: excessive verbal production; RVP: reduced verbal production), within-group comparisons yielded the following statistically significant differences: in the FXS group, RUT > EVP (*t* = 5.221; *p* = 0.003; *d* = 2.828; *r* = 0.816), VUT > EVP (*t* = 3.450; *p* = 0.018; *d* = 2.121; *r* = 0.727), and RVP > EVP (*t* = 2.492; *p* = 0.05; *d* = 1.293; *r* = 0.543) and in the WS group, RUT < VUT (*t* = −4.638; *p* = 0.006; *d* = −2.626; *r* = −0.795) and VUT > EVP (*t* = 4.600; *p* = 0.006; *d* = 3.092; *r* = 0.839).

In the types of violations of the MMN (REP: repetition of words; REF: syntactic reformulation; PER_F: perseveration of form; ORD: syntactic order), within-group comparisons yielded the following statistically significant differences: in the FXS group, REP > REF (*t* = 3.054; *p* = 0.028; *d* = 1.686; *r* = 0.644) and REP > ORD (*t* = 3.272; *p* = 0.022; *d* = 1.900; *r* = 0.688) and in the WS group, REP > ORD (*t* = 2.819; *p* = 0.037; *d* = 1.457; *r* = 0.588), REP > PER_F (*t* = 3.156; *p* = 0.025; *d* = 1.874; *r* = 0.683), REF > ORD (*t* = 2.718; *p* = 0.042; *d* = 2.262; *r* = 0.749), REF > PER_F (*t* = 5.923; *p* = 0.002; *d* = 5.099; *r* = 0.930), and ORD > PER_F (*t* = −3.212; *p* = 0.024; *d* = 1.581; *r* = 0.620).

**Figure 1:**
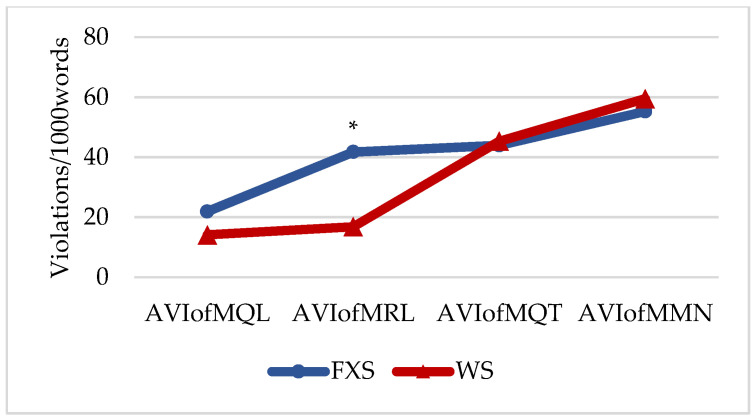
Profiles of aggregate violation indexes (AVIs) of maxims. Note: MQL = maxim of quality; MRL = maxim of relation; MQT = maxim of quantity; MMN = maxim of manner, * = *p* < 0.05.

Figure 1 shows the group profiles based on the AVIs of the cooperation maxims (MQL: maxim of quality; MRL: maxim of relation; MQT: maxim of quantity; MMN: maxim of manner). The profiles of both groups are comparable, except for the case of the MRL, where the individuals with FXS presented a higher frequency of non-related, tangential and, particularly, perseveration of topic. Furthermore, within-group differences in the FXS group were as follows: MQL < MRL (*t* = −5.049; *p* = 0.004; *d* = −1.131; *r* = −0.492); MQL < MQT (*t* = −2.687; *p* = 0.043; *d* = −1.168; *r* = −0.504); MQL < MMN (*t* = −2.566; *p* = 0.050; *d* = −1.923; *r* = −0.693). In the WS group, within-group differences were MQL < MQT (*t* = −4.084; *p* = 0.009; *d* = −2.713; *r* = −0.804), MQL < MMN (*t* = −4.769; *p* = 0.005; *d* = −2.913; *r* = −2.142), MRL < MQT (*t* = −3.851; *p* = 0.012; *d* = −2.641; *r* = −0.797), and MRL < MMN (*t* = −5.064; *p* = 0.004; *d* = −2.844; *r* = −0.818).

**Figure 2:**
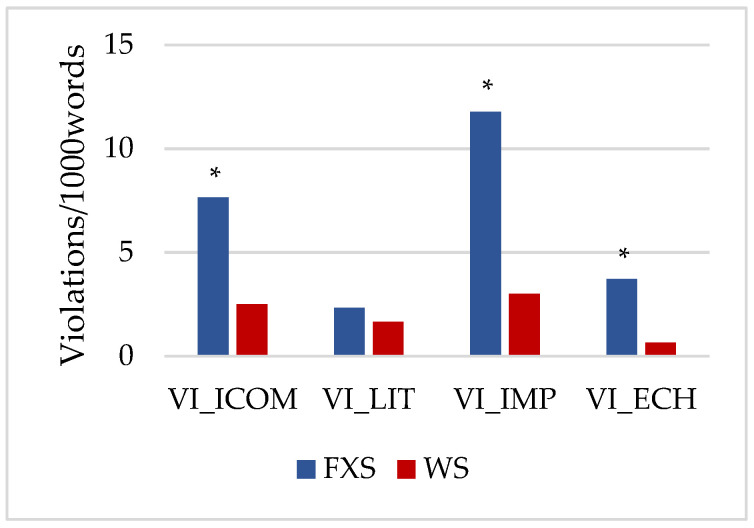
Violation indexes (VIs) of conversational response. Note: ICOM = incomprehension; LIT = literality; IMP = impulsivity; ECH = echolalia, * = *p* < 0.05.

Figure 2 shows the violation indexes (VIs) in the conversational responses for each group based on several characteristics of the responses (optional labels of the PREP-CORP): ICOM: incomprehension; LIT: literality; IMP: impulsivity; ECH: echolalia. As for between-group comparisons, significantly higher VIs were observed in the FXS group in ICOM (*t* = −2.352; *p* = 0.041), IMP (*t* = −2.694; *p* = 0.039), and ECH (*t* = −3.009; *p* = 0.013). Within-group differences were found only in the FXS group between ICOM > LIT (*t* = 3.330; *p* = 0.021; *d* = −0.667; *r* = −0.316) and IMP > LIT (*t* = 2.828; *p* = 0.037; *d* = −1.684; *r* = −0.644).

The sum of the VIs resulted in a global measure of conversational response (AVIofCONRES) for each syndrome: FXS group (mean = 25.47; SD = 10.71) and SW group (mean = 7.83; SD = 3.54). These differences were statistically significant (*t* = −3.827; *p* = 0.008; *d* = 2.44; *r* = 0.77).

## 4. Discussion

The purpose of the present study was to assess the profiles of pragmatic impairment in adults with FXS and WS, analyzing the violation of the Grice’s maxims of cooperation in spontaneous conversations based on the PREP-CORP protocol. Within- and between-syndrome differences in the types of violations of the maxims were analyzed.

An aggregate measure of overall observance of the conversational maxims indicated that the participants with FXS and WS violated to some extent all the cooperation maxims. This entailed difficulties with sharing information adapted to the social context or to the needs of the interlocutor and with following the conversational rules. The communicative quality of their questions, responses, and reports, as well as the contextual relevance of their interventions in the conversation, were compromised in several ways. The information provided tended to be vague, redundant, or reduced. Conversational production included a high rate of repetitions, as well as reformulations, perseverations of form, and alterations of the syntactic order. Previous research had already suggested the presence of alterations in the conversational discourse of people with FXS [14,37,38,39,40,42,43] and, to a lesser extent, also in people with WS [45,46,47]. Most of these characteristics were already included in the category of *Semantic-Pragmatic Syndrome without Autism* [17], but the present study revealed both within- and between-syndrome differences, suggesting that pragmatic impairment is not homogenous across syndromes.

As for the goal related to pragmatic assessment, PREP-CORP provided a feasible method for the pragmatic analysis of speech corpora, yielding results consistent with those of other methods [86]. Specifically, it allowed a detailed assessment of the main content characteristics and formal aspects of the conversational cooperation in adults with FXS and WS in natural settings, which is essential for evaluating pragmatic competence [77].

### 4.1. Within-Syndrome Differences in the Profiles of Conversational Cooperation

In the FXS group, high indexes of violation of the maxims of manner, quantity, and relation were observed. Repetition was the most salient type of violation of the maxim of manner. Results show a significantly higher frequency of repetition than of reformulation, in accordance with the differences observed by Belser and Sudhalter [14] between repetitive (i.e., repetitions of sounds, words, or sentences) and non-repetitive dysfluencies (i.e., self-corrections, reformulations, or false-starts). These authors interpreted the prevalence of perseveration and dysfluency in FXS as being associated with social anxiety and hyperarousal. Vague utterances followed by reduced verbal production were the most frequent types of violation of the maxim of quantity, while excessive verbal production was extraordinarily rare. Concerning the maxim of relation, topic perseveration predominated over tangential language. Conversational response showed high levels of impulsivity and incomprehension, together with a noticeable frequency of echolalia.

In the WS group, violations of the maxims of manner and quantity were significantly more frequent than those of the maxims of relation and quality. Repetition and reformulation were the most prevalent types of violation of the maxim of manner, while perseveration of form was less common. This is consistent with studies reporting dysfluency (hesitations, repetitions, and pauses) as a significant marker of the speech disorder of individuals with WS, which has been explained considering the cognitive and linguistic demands of the narrative and conversational tasks, in terms of language processing and planning [93]. Reduced verbal production followed by vague expression were the most frequent types of violation of the maxim of quantity. Although violations of the maxim of relation were less frequent, an important number of tangential utterances and perseverations of topic were observed, whereas non-related utterances occurred rarely. Inadequacy of conversational response was not prominent, with a particularly low incidence of echolalia.

### 4.2. Between-Syndrome Differences in the Profiles of Conversational Cooperation

Between-syndrome comparisons of the global index of violation of conversational cooperation in the FXS and the WS groups yielded no statistical differences. However, the present study found some similarities and a number of important differences in their profiles of pragmatic impairment. In both groups, violations of the maxims of quantity and manner were particularly high, while violations of the maxim of quality were less frequent. These findings may suggest an asynchrony between the pragmatic profiles and the underpinning lexical and grammatical skills. The inadequate use of language in conversation, with regard to the informative needs of the interlocutor, might not be consistent with the relative strengths observed in the lexical and grammatical levels, especially in WS [1,9,10,11]. In the case of the maxim of quantity, both groups showed redundant, vague, and reduced verbal production in similar proportion, in contrast with low indexes of excessive verbal production. This is consistent with socio-behavioral characteristics in FXS, such as selective mutism, social anxiety, and social phobia [94]. In contrast, people with WS were characterized in early descriptions as being loquacious or hyperverbal [3], although studies focusing specifically on the analysis of conversation underscored the insufficient quantity of information provided [46]. Violations of the maxim of manner presented a similar frequency, with a high proportion of repetitions and a lower proportion of alterations of syntactic order, but more differentiated cross-syndrome profiles.

The most striking difference between the profiles was a significantly higher index of violations of the maxim of relation in the adults with FXS, which indicates that their utterances tended to be more inappropriate or non-contingent. More specifically, regarding the contextual relevance, participants with FXS produced more tangential utterances and, above all, more topic perseverative utterances. These findings are consistent with the results of Roberts et al. [41] indicating that non-contingent and perseverative speech were the most salient features in boys with FXS compared with peers with Down syndrome. Despite the higher levels of perseveration and tangential language in the FXS group, these types of violation of the conversational maxims still accounted for more than 12% in the pragmatic profile of cooperation of the WS group. In relative terms, topic perseveration accounted for almost 54% of violations of the maxim of relation in the participants with FXS, while tangential language accounted for 40%; inversely, in the WS group, it accounted for almost 42% of violations of the maxim, while tangential language accounted for 54%. Thus, qualitative differences in the profiles were also observed, with a more marked tendency to perseveration in the FXS group and to tangential language in the WS group. Perseveration of topic and tangential language might then be considered not unique to FXS but also a symptom of pragmatic impairment in adults with WS, so that in this respect there would essentially exist a difference in degree between the syndromes. Social anxiety could account for these results, given that it has been described in both syndromes [3,14,15,93]. The relationship between anxiety and social communication could be mediated by executive functioning involved in language processing and planning during conversation and narration, although the existence of shared underlying mechanisms has also been questioned [15,93,95]. Behavioral inflexibility occurring across genetic and neurodevelopmental disorders has also been related to anxiety and social communication [96].

Despite the fact that violation of the maxim of manner reached similar levels in both groups, prominent differences were also observed concerning the types of violation. On the one hand, perseveration of form (i.e., phrase perseveration) was significantly higher in the FXS group, which confirms a great tendency to topic and phrase perseveration as the most salient feature of conversational discourse in people with FXS, as it had been observed in previous studies [40,41,43]. It has been suggested that utterance perseveration might be more distinctive in the FXS profile than topic perseveration [95]. The presence of such stereotyped and idiosyncratic language in adults with FXS and without autism would confirm that phrase and topic perseveration is part of the FXS phenotype [2] and, therefore, should not be considered among the discriminating criteria of ASD comorbidity in FXS [52]. On the other hand, the individuals in the WS group presented a significantly higher index of reformulation, which to our knowledge is an aspect not described previously in the literature. Such tendency to reformulate or rephrase during discourse construction in the case of individuals with WS may partly explain why their relative strengths in grammar are not matched by pragmatic skills in conversational use. In fact, the WS group showed a higher degree of repetition considering both repetitive and non-repetitive dysfluencies. These findings highlight the need to differentiate between perseveration and dysfluency (i.e., repetition) in the characterization of the pragmatic profiles of both syndromes.

### 4.3. Differences in the Profiles of Conversational Response

The analysis of the conversational response also revealed differences between the FXS and the WS groups, and it was significantly more impaired in the FXS group, also showing a more differentiated within-syndrome profile. In both syndromes, impulsivity and incomprehension were the more frequent characteristics of inadequate responses, accounting for more than 70% of the index, while participants also exhibited a tendency to an overly literal interpretation of utterances. Such pragmatic difficulties in processing syntax and semantics in context have been commonly observed in atypical populations, including FXS, WS, and ASD, and have been linked to theory of mind and metapragmatic deficits, which may reflect a failure to integrate a situation model including the speaker’s intent and the rest of the communicative context [97,98,99]. Qualitative differences in the profile come from a higher relative proportion of literal interpretation in the WS group and, conversely, a lower relative proportion of echolalia. Language comprehension during conversation might have specific characteristics of literality in people with WS, which would imply a relative weakness in line with the findings of previous studies [100,101].

The participants with FXS showed a much higher index of impulsivity, which is associated with ADHD and considered part of the core behavioral phenotype of the syndrome [102,103]. Responses reflecting immediacy or impulsivity reached the highest violation index within the aggregate index of conversational response in both groups. Impulsivity appears to have an effect on language comprehension and social competence in young adults with ADHD [104]; thus, it may also be partly responsible for the social communication disorder in people with FXS and WS.

Responses indicating incomprehension of the conversation topic were also significantly more frequent in the participants with FXS, which could suggest an area of weakness, as it was observed in early communication by Roberts et al. [105] using a test of receptive vocabulary. However, Abbeduto et al. [106] failed to replicate these results in adolescents and young adults with FXS who showed levels of comprehension expected for their mental age. In their study, scores in auditory comprehension were higher than those from matched peers with Down syndrome, which was consistent with the results from Price et al. [107] on language comprehension, comparing children and adolescents with Down syndrome and FXS. Although in the WS group the index of incomprehension was significantly lower, specific difficulties have been reported in children with WS regarding language comprehension [100]. Furthermore, Martin et al. [108] found that children with FXS without ASD were able to signal the incomprehension of confusing messages, while in a similar task, children with WS failed to verbalize the inadequacy of the messages more than half the time [101].

Echolalic responses were significantly higher in the FXS group, albeit far less frequent than self-repetitions, which is consistent with previous studies indicating a profile of low proportion of echolalia in individuals with FXS vs. high proportion in ASD [37,109]. The analysis of echolalia may then contribute to differentiation of the profiles of neurodevelopmental disorders, as echolalia does not appear to be simply a manifestation of general intellectual impairment [39,110].

### 4.4. Clinical Implications

The results of the present study show that individuals with FXS and WS without a diagnosis of ASD may exhibit different degrees and profiles of pragmatic impairment [20,86], and therefore they could meet some diagnostic criteria of Social (Pragmatic) Communication Disorder (SPCD). These findings are thus consistent with previous studies suggesting partial overlap and a high degree of comorbidity of the SPCD diagnosis with other neurodevelopmental disorders [24,25]. In the same vein, the results confirm that pragmatic impairment may not necessarily be a symptom exclusive to autism [19]. A diagnosis of comorbid ASD based only on the degree of pragmatic impairment might be inaccurate and misleading for assessment and intervention in cases of intellectual disability [61,62], particularly in those with genetic syndromes [72]. Furthermore, a narrow conception of SPCD would also pose therapeutic dilemmas since, in FXS and WS, cross-cutting needs for assessment and treatment of social communication difficulties would persist [23,48,68,111]. Pragmatic language skills contribute to the capacity for adult independent functioning in FXS [112]; therefore, language intervention may be essential for this purpose. Recent studies from a transdiagnostic perspective have shown the feasibility and effectiveness of therapeutic interventions on socio-communicative skills, such as conversational responsivity, in individuals with FXS, WS, and ASD [113,114].

It is important to acknowledge several limitations of the present study. The sample size was small and did not control for gender differences, for which reason the objectives were defined as exploratory. The consequent lack of statistical power and of adjustment for multiple comparisons may have influenced the assessment of differences. However, the conversational sample sizes were adequate and could have partially compensated for this limitation, as can be noted in the effect sizes. Different settings in which the speech samples were taken (home for WS, work for FXS) and the very different age ranges may have affected the results. The focus on group means’ differences and similarities rather than individual differences is also a source of limitations. Stojanovik et al. [115] found striking individual differences in standardized linguistic measures and in conversational settings in a small sample of children with WS. The inclusion of a control group of adults with TD could have provided further results, although they would have been expected to observe conversational maxims. Correlations of cognitive abilities and linguistic measures observed in previous studies [116] suggest that the lack of mental and verbal age controls in the present study may have prevented ruling out the effect of those variables on the differences between the groups. Elicitation context should include in future studies not only conversational but also narrative tasks, as they imply differences in language complexity. Finally, the variation in definitions of perseveration, repetition, or disfluency in the literature makes it difficult to compare the results, as pointed out by Murphy and Abbeduto [12], and therefore it would be necessary to discuss in more detail the coding provided by the PREP-CORP.

## 5. Conclusions

Adult participants with FXS and WS in the present study showed violations of all the maxims of conversation to varying degrees, which may have an impact on their pragmatic skills. The profiles of cooperation resulting from the assessment of such pragmatic impairment using the PREP-CORP shared some characteristics, including reduced verbal production (maxim of quantity), repetitive dysfluencies (maxim of manner), and similar degree of violation of the maxim of quality. Syndrome-specific features were also observed: in the FXS profile, higher proportion of non-contingent language (maxim of relation) and perseveration of topic and form (maxims of relation and manner); in the WS profile, higher proportion of reformulation (maxim of manner). Conversational response showed increased levels of impulsivity, incomprehension, and echolalia in FXS. Thus, both profiles presented different degrees and forms of pragmatic impairment in the absence of a diagnosis of comorbid ASD, which raises the need for assessment and intervention methods that specifically address the social communication skills of adults with FXS and WS.

## Figures and Tables

**Table 1:** Differences in the violation indexes (VIs, AVIs) of the maxims (M).

	FXS-G Mean (SD)	WS-G Mean (SD)	*t*	*p*	*d*	*r*
**AVIofMQL**	21.91 (15.52)	14.16 (6.3)	1.132	0.297	0.6543	0.3109
**AVIofMRL** VI_NRL VI_TNG VI_PER_T	41.75 (20.18) 3.61 (3.73) 15.68 (8.95) 22.46 (11.73)	16.83 (4.30) 0.66 (0.81) 9.16 (3.19) 7.00 (4.14)	2.958 1.885 1.678 3.045	**0.028** * 0.113 0.142 **0.022** *	1.7080 1.0930 0.9768 1.7576	0.6494 0.4795 0.4388 0.6601
**AVIofMQT** VI_RUT VI_VUT VI_EVP VI_RVP	43.94 (22.25) 8.52 (4.17) 18.53 (12.8) 0.21 (0.33) 16.67 (16.23)	45.33 (15.92) 6.83 (2.64) 16.66 (5.68) 2.33 (4.80) 19.50 (16.30)	0.124 0.840 0.327 −1.079 0.301	0.904 0.420 0.754 0.306 0.770	−0.0718 0.6324 0.2175 −0.6231 −0.1875	−0.0359 0.3015 0.1081 −0.2974 −0.0933
**AVIofMMN** VI_REP VI_REF VI_PER_F VI_ORD	55.28 (20.07) 24.84 (12.60) 8.76 (6.79) 14.08 (8.00) 7.59 (4.08)	59.50 (21.82) 28.0 (18.77) 17.5 (3.93) 4.66 (2.25) 9.33 (4.41)	0.348 −0.342 −2.725 2.773 −0.707	0.735 0.739 **0.021** * 0.020 * 0.496	−0.1950 −0.2614 −1.8973 1.7149 −0.5000	−0.0970 −0.1296 −0.6882 0.6509 −0.2425
**AVIofCOOP**	162.88 (43.92)	135.16 (24.49)	−1.350	0.207	0.7753	0.3614

Note: **AVI** = aggregate violation indexes, VI = violation indexes, **MQL** = maxim of quality, **MRL** = maxim of relation, **MQT** = maxim of quantity, **MMN** = maxim of manner, **COOP** = cooperation, NRL = non-related, TNG = tangential, PER_T = perseveration of topic, RUT = redundant, VUT = vague, EVP = excessive verbal production, RVP = reduced verbal production, REP = repetition of words, REF = reformulation, PER_F = perseveration of form, ORD = syntactic order, * = *p* < 0.05.

## Data Availability

The data presented in this study are available on request from the corresponding author.

## Appendix A

**Table A1:** PREP-CORP Pragmatic Evaluation Protocol for Corpora.

Enunciative Pragmatics %xepr Inferences Sublevel $i				
PREP Item	Main Label	Secondary Label 1	Secondary Label 2	Optional Label
5. Cooperative Principle: Maxims $i5	Maxim of Quality MQL	Question QST	World Reality WOR	Impulsivity IMP Inadequate Comprehension ICOM Literal Language LIT Perseveration PER Echolalia ECH Fabulation FAB Theory of Mind TOM
		Answer ASW		
		Report RPT		
	Maxim of Quantity MQT	Redundant Utterances RUT		
		Vague Utterances VUT		
		Excessive Verbal Production EVP		
		Reduced Verbal Production RVP	Onomatopoeia ONO	
			Interjection INT	
	Maxim of Relation MRL	QST	Non-related NRL Tangential TNG Perseveration (of topic) PER	
		ASW		
		RPT		
	Maxim of Manner MMN	Repetition REP		
		Reformulation REF		
		Perseveration PER		
		Word Order ORD	Addition ADI	
			Omission OMI	
			Inversion INV	
			Substitution SST	
	Particularized Implicatures PIM	Flouting MQL PMQL	In/Adequate Comprehension ACOM/ICOM In/Adequate Production ADQ/IDQ	
		Flouting MQT PMQT		
		Flouting MRL PMRL		
		Flouting MMN PMMN		

Note: extract from PREP-CORP Pragmatic Evaluation Protocol [79], section of enunciative pragmatics.
